# Thinking Outside the Block: External Focus of Attention Improves Reaction Times and Movement Preparation Times in Collegiate Track Sprinters

**DOI:** 10.3390/sports6040120

**Published:** 2018-10-19

**Authors:** Attila J. Kovacs, Garrett F. Miles, Harsimran S. Baweja

**Affiliations:** 1 Department of Exercise and Sport Science, University of Wisconsin-La Crosse, La Crosse, WI 54601, USA; miles.garr@uwlax.edu; 2 Doctor of Physical Therapy Program, School of Exercise and Nutritional Sciences, San Diego State University, San Diego, CA 92119, USA; hbaweja@sdsu.edu

**Keywords:** motor control strategies, attentional focus, electromyography, reaction time, muscle activation

## Abstract

While focusing attention on external cues (EF) has been shown to enhance performance track and field coaches tend to provide instructions that promote internal focus of attention (IF) during block starts. The aims of this study were to determine: (1) whether promoting EF versus IF would improve reaction time (RT) of sprinters, and (2) if changes occur at the level of central processes during movement preparation (premotor RT) or peripheral processes during movement execution (motor RT). Twelve collegiate track sprinters (age 20.8 ± 1.7) completed three testing sessions under EF, IF, and no focus instruction (NF) conditions. RT was recorded from the left and right blocks. Muscle activation time (EMG) was recorded from the vastus lateralis and gastrocnemius muscles. Mean rear foot RT was significantly shorter (*p* < 0.0001) under the EF (212.11 ms) compared with the IF (234.21 ms) and NF conditions (236.87 ms). Front foot RT was significantly shorter (*p* < 0.05) during EF (250.24 ms), compared to IF (266.98 ms) but not shorter than the NF (268.73 ms) condition. Mean premotor RT under the EF condition (157.75 ms) was significantly shorter (*p* < 0.001) compared with the IF (181.90 ms) and NF (173.60 ms) conditions. No differences were found in motor RT across conditions (*p* > 0.05). Adopting an EF improves RT during sprint starts. This improvement likely originates from a shortening in movement preparation time, as opposed to a faster excitation contraction coupling of the muscle fibers. These findings could potentially contribute to the development of new coaching methods aimed at improving the starting technique of athletes.

## 1. Introduction

The start of a short distance running race can have profound effects on the outcome, accounting for approximately 5% of total race time in the 100 m dash [1]. The goal is to accelerate from the starting block in a linear direction as quickly as possible following the reaction to a gunshot, allowing the athlete to reach maximum running velocity as quickly as possible. Therefore, integration of any variable that reduces race time has the potential to benefit these athletes. In addition to biomechanical and physiological factors, the efficacy of information processes involved in movement preparation and execution could influence the production speed of complex tasks that require a quick response to a stimulus and the coordination of multiple effectors [2]. The focus of a performer’s attention may be one such factor that could enhance reaction time (RT) during the start of a sprint race.

Two distinct forms of attentional focus that can influence movement output performance have been identified: external (EF) and internal (IF) focus of attention [3]. An EF requires focusing on the intended movement effect on an implement or the environment, whereas an IF requires the performer to focus on their own body movements [3]. Over the last two decades, a considerable amount of research has demonstrated that an EF, compared to an IF has beneficial effects on motor learning and performance. These benefits have been seen in a multitude of tasks including dynamic balance tasks [4,5], throwing [6], golf shots [7,8], soccer kicks [9], baseball batting [10], bimanual coordination [11], accurate force production [12,13], vertical jumping [14], and standing long jump [15], as well as others.

The effects these different attentional foci have on motor learning and performance have been explained by the constrained action hypothesis [3]. According to this hypothesis, an EF facilitates the automatic self-organization of movements whereas an IF produces conscious interference of those otherwise automatic control processes. Automaticity is associated with greater economy in movement production and motor unit recruitment, as revealed by reduced neuromuscular activity and different mean power frequency profiles when utilizing an EF compared to IF [16].

Reaction time is a measure of the time from the arrival of a stimulus to the beginning of the response to it. This measure can become longer or shorter depending on the number of stimuli that could be presented, or the number of choices that can be made depending on those stimuli. The fewer the number of stimuli and the number of possible associated responses, the shorter the RT. According to Jensen [17], simple reaction time (SRT) latencies provide one of the most objective metrics for comparing processing speed. During a SRT task, there is only one appropriate response to a known stimulus and, as such, the shortest RTs occur during this type of task [18,19]. For example, during a track sprint start, leaving the blocks as fast as possible is the only appropriate desired response to the firing of a starting gun. Shorter RTs have been shown under an EF, compared to an IF, during a track sprint start, and it has been proposed that these improvements might be due to a more efficient movement preparation process [2]. Additionally, a SRT task encompasses both central and peripheral mechanisms that lead to the production of a response to the stimulus. It is uncertain whether these changes in RT occur during the stages of central processing of information, or through peripheral mechanisms associated with the conduction of the electrical impulse and/or the contraction of the muscles.

The central and peripheral events that occur during a RT task can be partitioned into premotor and motor RT within the RT paradigm [20,21]. Premotor RT is defined as the time interval elapsed from the presentation of the stimulus to the first change in electromyography (EMG) activity of the muscle, while motor RT is the time interval elapsed between the first change in EMG activity, and the initiation of force production [20,21,22]. Premotor RT is associated with the stages of information processing which includes stimulus detection, response selection, and response programing, while motor RT is associated with the excitation contraction coupling of the muscle fibers [21]. Although there is conflicting evidence of selective recruitment or reversal of size principle during ballistic movement, maximum shortening velocity of the muscle is determined by the fastest fibers [23,24]. Therefore, during movement that requires maximum power production, the active fibers should be the same resulting in the same speed of excitation contraction coupling (i.e., time to reach peak force) if the movement were repeated without fatigue [23]. The purpose of the present study was to determine whether instructions promoting external versus internal focus of attention would influence simple reaction time during a track sprint start task. More importantly, of primary interest was to determine *if focus of attention manipulation would influence primarily central processes during movement preparation*, or peripheral processes during movement execution. By distinguishing premotor and motor RTs within an SRT task, it is possible to assume that any changes observed within premotor RT, with no change in motor RT, are occurring as a result of changes in central processing speed. Furthermore, by using an SRT task in which the stimulus is known, and there is only one appropriate response, any changes in premotor RT are likely occurring within the response programing stage of information processing. Thus, our prediction was that RT would be shorter under EF as compared with IF, and if attentional focus influences primarily central processing, the premotor RT would be longer under IF compared to an EF condition, with no differences in motor RT or in the time required for the muscle to produce peak levels of force.

## 2. Materials and Methods

### 2.1. Participants

Twelve Division III collegiate track sprinters (age 20.8 ± 1.7) with several years of experience in sprinting (5.3 ± 1.02) volunteered to participate in the study (*n* = 8 women, *n* = 4 men). Participants were familiar with the task, but naïve to the purpose of the study. Informed consent was obtained from participants before testing began. All forms and experimental methods were approved by the University of Wisconsin-La Crosse Institutional Review Board.

### 2.2. Apparatus

Sprint starts were conducted on a synthetic indoor track surface. Force data were acquired for the front and rear foot via two force plate sensors (FP3, Biometrics Ltd., Newport, UK) mounted to a standard track starting block. Electromyographic data were acquired through a Delsys Trigno^TM^ (Natick, MA, USA) wireless system from the left and right vastus lateralis (VL), as well as the left and right medial gastrocnemius (GM). These muscles have previously been identified as prime movers during a block sprint start [25]. The force and EMG data were time synchronized through an A/D board (USB-6210, National Instruments, Austin, TX, USA), sampled at 2000 Hz, and stored on a personal computer (Latitude E6530, Dell, Round Rock, TX, USA) for further analysis. The start signal was produced by a gunshot sound clip (44,100 Hz; 16 bits/sample), and played through external speakers (Labtec LCS-1030, Logitech, Newark, CA, USA) placed behind the starting block. Data acquisition and processing was performed through a custom developed computer program (Matlab, Mathworks, Natick, MA, USA).

### 2.3. Procedure

Three separate sessions were used to collect data under three different focus conditions: no focus (NF), external focus (EF), and internal focus (IF). Participants were instructed to “focus on pushing the blocks away” during the EF condition, to “focus on extending your knees” during the IF condition, while no additional instructions were given during the NF condition. Test sessions were separated by a minimum of two days. Fifteen starts were performed each session, with ~1–2 min of rest between each trial. Participants performed their routine warm-up before each test session as they would before a competitive sprint event. During the experimental task, they were instructed to accelerate as fast as possible to a line placed 6 m from the start line. This distance was chosen in order to give the athletes a distance goal, while also minimizing fatigue that could occur from running longer distances. Before the trials, participants were informed that they would receive the start commands “on your mark” and “set” before hearing the gunshot. The focus cues were given following the “on your mark” command, before the “set” command and gunshot. The first session always consisted of the NF condition, while the EF and IF conditions were completed utilizing a counterbalanced within-participant design. Participants were not given any type of feedback relative to their performance during the sessions.

### 2.4. Signal Processing

The force signal was filtered using a second order low-pass Butterworth filter with cut-off frequency at 15 Hz and zero phase-shift. Similarly, to obtain the linear envelope, the rectified EMG signal was filtered with a cut-off frequency of 35 Hz.

**Figure 1:**
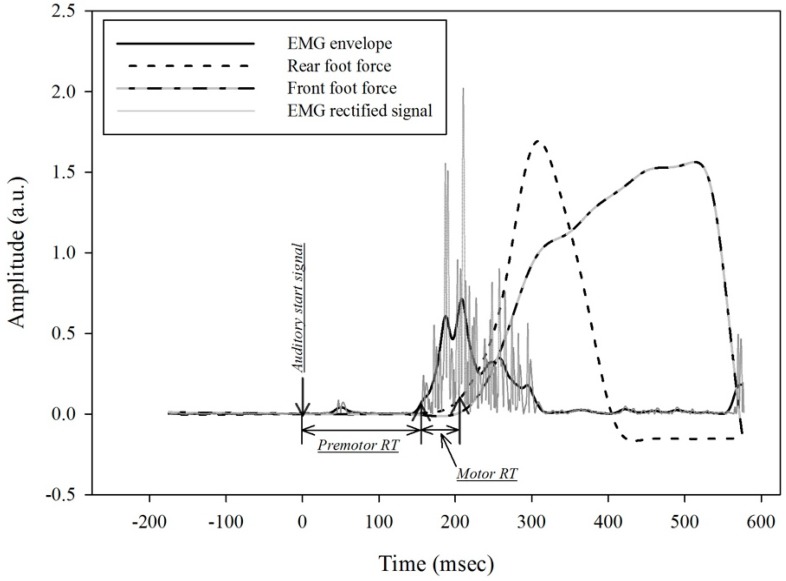
Example of the electromyography (EMG) signal processing and determining the time frame for the dependent variables for one trial.

The time elapsed between the auditory signal and the detection of a 5% change relative to the maximum force applied against the block was determined as the RT. Premotor RT was determined as the time elapsed between the auditory signal and the detection of a 5% change relative to maximum in the EMG envelope, while Motor RT as the time elapsed between the occurrence of a 5% change relative to maximum in the EMG envelope and a 5% change relative to the maximum force applied against the block. (Figure 1). Time to reach peak force was determined as the interval between a 5% change in force and reaching peak force production against the block.

### 2.5. Statistical Analysis

In order to detect and eliminate outliers before further analysis, absolute deviation around the median was used [26,27]. Whenever a value exceeded the conservative criteria of ±3 Median Absolute Deviation, the trial was eliminated from further analysis. A preliminary analysis, separating the total trials within each focus condition into three groups (trials 1–5, 6–10, and 11–15), was used to detect any potential changes in RT due to fatigue over the course of fifteen trials. A one-way ANOVA with repeated measures on groups failed to detect any differences, indicating no fatigue occurred over the course of the testing. Therefore, all trials within a focus condition were grouped together for further analyses.

All dependent variables were analyzed using a one-way ANOVA with repeated measures on focus factor (NF, IF, and EF), and Bonferroni adjustments for multiple comparisons. Criterion for significance level was set using an α level (*p* < 0.05). Data are presented as means and standard error of the means (SE) in the text, figures, and table.

## 3. Results

### 3.1. Fatigue

There were no differences in rear foot RT across groups of trials from the beginning and end of the testing session for the NF (F_(2,22)_ = 0.485, *p* = 0.622), IF (F_(2,22)_ = 0.135, *p* = 0.874), and EF (F_(2,22)_ = 0.343, *p* = 0.713) conditions. Therefore, all trials within a focus condition were grouped together for further analyses.

### 3.2. Reaction Time

**Figure 2:**
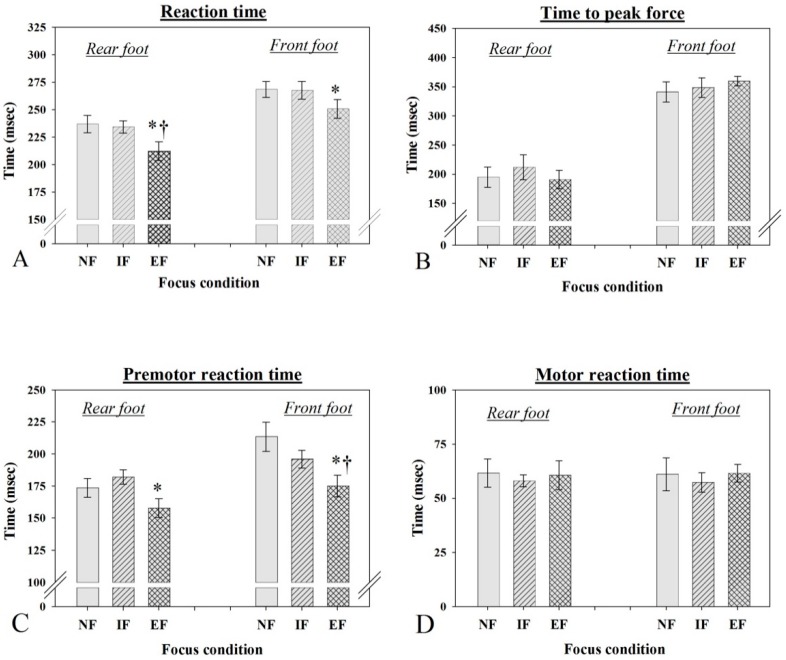
Mean duration under the no (NF), internal (IF) and external (EF) focus conditions for reaction time (**A**); time to peak force (**B**); pre-motor reaction time (**C**); and motor reaction time (**D**). Error bars represent standard error of the mean (SE). * Significantly less than IF (*p* < 0.05). ^†^ Significantly less than NF (*p* < 0.05).

There was a significant main effect of focus condition on rear foot RT F_(2,22)_ = 14.996, *p* < 0.0001, η_p_^2^ = 0.57, post hoc tests revealed that rear foot RT during the EF condition (M = 212.11 ms, SE = 8.45) was significantly shorter than both the IF (M = 234.21 ms, SE = 5.76) and NF conditions (M = 236.87 ms, SE = 8.82) (*p* < 0.002), with the last two not being different from one another (*p* > 0.05) (Figure 2). Front foot RT was also found to be significantly different between focus conditions, F_(2,22)_ = 4.541, *p* < 0.05, η_p_^2^ = 0.29, with further analysis revealing that front foot RT during the EF condition (M = 250.24 ms, SE = 17.24) was significantly shorter than IF, *p* < 0.05 (M = 266.98 ms, SE = 16.44) but not NF (M = 268.73 ms, SE = 14.23) (*p* < 0.05). No difference in front foot RT was found between IF and NF conditions (*p* > 0.05) (Figure 2A).

### 3.3. Time to Peak Force

No significant differences were found between the various focus conditions in rear foot and front foot time to peak force (Figure 2B).

### 3.4. Electromyography

The rear foot Vastus Lateralis (VL) was the first muscle activated during the sprint start task, therefore premotor RT was determined as the time elapsed between the gunshot and the activation of the rear foot VL. Motor RT was determined as the time interval between rear foot VL activation and the initiation of force production (Figure 1).

#### 3.4.1. Pre-Motor Reaction Time

There was a significant main effect of focus condition on the rear foot VL activation time, F_(2,22)_ = 10.541, *p* < 0.001,η_p_^2^ = 0.49. Further analysis revealed that the rear leg VL was activated earlier during the EF condition (M = 157.75 ms, SE = 7.38) compared to the IF condition (M = 181.90 ms, SE = 5.72) but not significantly earlier when compared to the NF condition (M = 173.60 ms, SE = 7.30) (Figure 2C). No difference was found in rear VL activation time between IF and NF conditions (*p* > 0.05). Similarly, front leg VL EMG activation was significantly different between focus conditions, F_(2,22)_ = 9.228, *p* < 0.001, η_p_^2^ = 0.46, post hoc tests revealed that activation of the front foot VL during the EF condition (M = 174.90 ms, SE = 8.42) occurred significantly earlier than during IF (M = 195.98 ms, SE = 6.93), and the NF conditions (M = 213.42 ms, SE = 11.43). No difference was found in front VL activation time between IF and NF conditions (*p* > 0.05). Additionally, the main effect of focus condition was not significant in rear and front leg GM activation time (*p* > 0.05).

#### 3.4.2. Motor Reaction Time

There was no significant main effect of focus condition on time from VL activation to force initiation between EF, IF, and NF conditions for both the rear and front legs (*p* > 0.05).

A summary of the results can be found in Table 1.

## 4. Discussion

The aims of the present study were to determine whether instructions promoting external versus internal focus of attention would influence reaction time during a track sprint start, and to determine if these manipulations would influence primarily central processes during movement preparation, or peripheral processes during movement execution. In accordance with our hypothesis, RT was observed to be significantly shorter under an EF compared to the IF and NF conditions. More importantly, pre-motor RT was found to be significantly shorter under an EF condition compared to both IF and NF, while no changes in motor RT or the time to peak force were observed between focus conditions. The present experiment corroborates previous findings showing faster RT under EF during track sprint starts [2], or isometric force production [12], but to the best of our knowledge it is the first time when direct electrophysiological evidence is provided to demonstrate the notion that focus of attention manipulation can influence movement planning time.

In a RT task, pre-movement time is thought of as the time required to execute a whole set of processes associated with identifying a specific stimulus, choosing an appropriate response, planning the movement parameters, and in general does not dissociate between these processes. In a SRT task such as the one used in the current study, the stimulus and the response associated with it being known a priori, pre-movement time should mostly represent the stages of processing associated with programming a motor response [28].

In the present experiment, the pre-movement time was further partitioned into two separate stages: pre-motor RT and motor RT. This method allowed for a better differentiation of the true time costs of central processing (pre-motor RT) versus the time costs associated with activating the peripheral mechanisms responsible for movement production such as excitation-contraction coupling of the muscle fibers (motor RT). The attenuation of muscle activation time (pre-motor RT), as assessed by EMG, indicates that under the EF condition the motor commands reached the muscle earlier as opposed to the IF or NF condition. In other words, it seems that movement planning as a process is much more efficient and rapid when one directs the focus of attention externally. These finding are in line with previous experiments suggesting that RT increases with movement complexity [29]. The track sprint start is a complex whole-body sequential movement engaging many components (muscles). Therefore, the time required to prepare and initiate these components is increased when compared to a less complex SRT task. This is particularly evident when learning a novel complex task, where each element of a sequence is processed individually, from identifying a stimulus to selecting and executing the appropriate response [30]. With practice however, these individual components are organized and chunked together in bigger but fewer elements, with each one of these chunks being processed as a single unit, thus functionally reducing the amount of processing time required for movement planning. This leads not only to a more rapid, but also to a more coordinated and harmonic (less jerky) movement execution [30]. Klapp and Jagacinsky [29] showed that a simple RT to perform a sequence of movements increases with the number of chunks comprised in the sequence. In the present experiment, participants had several years of experience performing the sprint start, and presumably have developed a chunking pattern that allowed them to be proficient with the task. While in the present experiment the chunking pattern of the whole-body movement was not assessed, it is plausible to imagine that instructions specifically directing participant’s conscious attention towards an internal focus might have caused a disruption of the regular chunking pattern and movement sequence, and might have forced them to process multiple motor chunks, thus increasing the time required to process them. For example, through years of practice an athlete might hypothetically have developed two motor chunks, one to activate all the upper body and upper limb muscles involved in the sequence, and another for all the lower body muscles (i.e., hip-knee-ankle extensors). Consciously directing attention towards a very specific component of the movement sequence (extending their knees/internal focus), might have led to the segmentation of the latter chunk in two distinct parts. This would result in increased time during movement preparation, requiring the processing of two new smaller chunks (hip extensors and knee-ankle extensors), as opposed to one more complex chunk.

Alternatively, it is possible that the participants might have shifted to using a different strategy of muscle recruitment altogether. Using electrophysiological measures, Lohse et al. [13] and Wulf et al. [14] suggest that the way motor units are recruited under IF relative to EF is less efficient due to increased co-contraction between agonist and antagonist muscle pairs. In the present experiment, the electrophysiological activity of the antagonist muscles was not recorded. However, it is possible that the observed increase in the time needed to initiate muscle activation (premotor RT) under the IF condition, was due to the additional processing of parameters needed to control the activity of the antagonist muscles.

As was hypothesized, the duration of the motor RT, defined as the time elapsed between the detection of muscle activation and the initiation of force production, was similar under the different focus conditions. This time interval is thought of as representing the initial excitation-contraction coupling at the muscle level. Our results suggest that these processes were not influenced by the type of focus adopted by participants. Moreover, the rate of force production, as illustrated by the time to peak force, was also similar under the different focus conditions indicating that the muscles were contracting at the same velocity regardless which type of attentional focus was adopted. These results indicate that all peripheral mechanisms related to the contraction of the muscles, occurred similarly across the different conditions, further suggesting that any improvements in RT under the EF condition were due to a shortening of the time required for the central processes to occur.

Contrary to our predictions, there was no difference between focus conditions on the activation time of the GM muscles. Overall participants seemed to activate the GM muscles faster under the EF condition when compared with the IF and NF conditions, but this difference was found not to be significant. In addition, participants seemed to time the activation of the GM muscles after force production was initiated at the block level. In other words, the initial excitation-contraction of the VL muscles had produced levels of force that met our threshold criteria for detection (5% of peak force). Only then was the GM activated, and contributed to the overall force production. However, when looking at the individual data of the participants, we found two distinct relative timing patterns of muscle activation between participants. The majority of them (*n* = 9) showed a VL-force-GM sequence as detected according to our threshold values, while a subgroup of participants (*n* = 3) produced a VL-GM-force sequence. The track sprint start is a rapid, discrete, whole body movement whereby athletes need to rely on a motor program that has all parameters specified before movement initiation [22]. What the above data suggests is that various athletes might have developed different relative sequences, force and timing structures for their motor program during sprint starts. In other words, the kinetic chain for these athletes might be different, even when performing the same task. This raises the question whether there is an optimal kinetic chain during sprint starts that would then translate into producing higher levels of force in a shorter amount of time, thus propelling them out of the blocks faster? Although a faster RT does not always translate into a faster block clearance time, future studies need to determine if a certain relative structure of sequence, force and timing would lead to a more efficient kinetic chain, and thus an improved sprint start [2].

### Practical Applications

A survey conducted at the USA Track and Field Outdoor National Championships determined that 84.6% of track and field coaches use cues that instruct athletes to focus internally and that 69.2% of the athletes utilize internal focus during competition [31]. With no additional instructions given, the athletes in the current study likely self-selected to focus internally during the NF condition. This possibly explains why longer and similar premotor times were observed under the NF as well as the IF condition compared to the EF condition. By utilizing EF cues, track and field coaches may be able to further enhance their athletes sprint performance. This effect may be even more important for developing sprinters. In addition to external focus being effective with motor learning, use of external cues early on in an athlete’s development may promote self-selecting external focuses when the athletes are unable to receive cues from a coach.

Previous studies using tasks whereby efficiency relies on the capability to produce high levels of power, such as standing long jump or vertical jump, have shown that adopting EF as opposed to IF improves performance [14,15]. Although in the present study the absolute amount of force produced under the different focus conditions was not assessed, it would be interesting to see in the future whether a faster RT under EF conditions would also translate into an increased level of force produced. If that is the case, and given that the present study shows that time to peak force was quite similar under the various focus conditions, this would suggest that the amount of power produced by the athletes would also be different.

## 5. Conclusions

In summary, the type of attentional focus adopted has a significant effect on the participant’s reaction time and significant effect on movement preparation time. The present experiment provides important new electrophysiological evidence that suggests that adopting an external focus of attention does indeed reduce movement preparation time. External focus instructions led to significantly faster pre-motor RT, suggesting that central processes associated with movement planning have been carried out more efficiently. Motor RT did not differ across the various focuses of attention conditions, indicating that peripheral mechanisms responsible for muscle excitation-contraction coupling and contraction velocity are not affected by focus of attention manipulations.

The results from this study are consistent with previous findings in motor control, which suggests that explicitly attending to one’s own body mechanics leads to less effective movement outcomes. It appears that adopting different types of attentional focus interferes with the efficiency of the movement planning processes, even when the uncertainty of the stimulus-response association is reduced, such as during a simple reaction time task.

## Figures and Tables

**Table 1:** Mean duration (ms) under the no (NF), internal (IF) and external (EF) focus conditions. RT (reaction time), VL (Vastus Lateralis), GM (Gastrocnemius Medialis), EMG (Electromyography). Standard error in parentheses (SE).

	EF	IF	NF
**Rear foot**			
Premotor RT (VL EMG)	157.75 (7.38) *	181.90 (5.72)	173.60 (7.30)
Motor RT	60.67 (6.61)	58.04 (2.82)	61.61 (6.57)
RT	212.11 (8.45) *^,†^	234.21 (5.76)	236.87 (8.82)
Time to peak force	190.80 (15.81)	211.79 (21.50)	195.09 (17.24)
GM EMG	240.90 (15.68)	260.40 (14.02)	249.21 (12.06)
**Front foot**			
Premotor RT (VL EMG)	174.90 (8.42) *^,†^	195.98 (6.93)	213.42 (11.43)
Motor RT	61.50 (4.06)	57.30 (4.50)	61.03 (7.60)
RT	250.24 (17.24) *	266.98 (16.44)	268.73 (14.23)
Time to peak force	359.89 (8.28)	348.55 (16.87)	340.96 (17.54)
GM EMG	282.85 (25.31)	286.58 (20.69)	289.77 (18.10)

* Significantly less than IF (*p* < 0.05). ^†^ Significantly less than NF (*p* < 0.05).

## References

[1] Harland M.J., Steele J.R. (1997). Biomechanics of the sprint start. Sports Med..

[2] Ille A., Selin I., Do M., Thon B. (2013). Attentional focus effects on sprint start performance as a function of skill level. J. Sports Sci..

[3] Wulf G., McNevin N., Shea C.H. (2001). The automaticity of complex motor skill learning as a function of attentional focus. Q. J. Exp. Psychol..

[4] Jackson B.H., Holmes A.M. (2011). The effects of focus of attention and task objective consistency on learning a balance task. Res. Q. Exerc. Sport.

[5] Shea C.H., Wulf G. (1999). Enhancing motor learning through external focus instructions and feedback. Hum. Mov. Sci..

[6] Southard D. (2011). Attentional focus and control parameter: Effect on throwing pattern and performance. Res. Q. Exerc. Sport.

[7] Bell J.J., Hardy J. (2009). Effects of attentional focus on skilled performance in golf. J. Appl. Sport Psychol..

[8] Wulf G., Lauterbach B., Toole T. (1999). The learning advantages of an external focus of attention in golf. Res. Q. Exerc. Sport.

[9] Wulf G., McConnel N., Gärtner M., Schwarz A. (2002). Enhancing the learning of sport skills through external-focus feedback. J. Mot. Behav..

[10] Castaneda B., Gray R. (2007). Effects of focus of attention on baseball batting performance in players of different skill levels. J. Sport Exerc. Psychol..

[11] Hodges N., Franks I.M. (2000). Attention focusing instructions and coordination bias: Implications for learning a novel bimanual task. Hum. Mov. Sci..

[12] Lohse K.R. (2012). The influence of attention on learning and performance: Pre-movement time and accuracy in an isometric force production task. Hum. Mov. Sci..

[13] Lohse K.R., Sherwood D.E., Healy A.F. (2011). Neuromuscular Effects of Shifting the Focus of Attention in a Simple Force Production Task. J. Mot. Behav..

[14] Wulf G., Dufek J.S., Lozano L., Pettigrew C. (2010). Increased jump height and reduced EMG activity with and external focus of attention. Hum. Mov. Sci..

[15] Wu W.F.W., Porter J.M., Brown L.E. (2012). Effect of attentional focus strategies on peak force and performance in the standing long jump. J. Strength Cond. Res..

[16] Vance J., Wulf G., Tollner T., McNevin N., Mercer J. (2004). EMG activity as a function of the performer’s focus of attention. J. Mot. Behav..

[17] Jensen A.R. (2011). The theory of intelligence and its measurement. Intelligence.

[18] Hick W.E. (1952). On the rate of gain of information. Q. J. Exp. Psychol..

[19] Hyman R. (1953). Stimulus information as a determinant of reaction time. J. Exp. Psychol..

[20] Devita M., Montemurro S., Zangrossi A., Ramponi S., Marvisi M., Villani D., Mondini S. (2017). Cognitive and motor reaction times in obstructive sleep apnea syndrome: A study based on computerized measures. Brain Cogn..

[21] Szpala A., Rutkowska-Kucharska A. (2017). Electromechanical response times in the knee muscles in young and old women. Muscle Nerve.

[22] Schmidt R.A., Lee T.D. (2011). Motor Control and Learning: A Behavioral Emphasis.

[23] MacDougall J.D., Sale G.D. (2014). The Physiology of Training for High Performance.

[24] Josephson R.K., Edman K.A. (1988). The consequences of fiber heterogeneity on the force-velocity relation of skeletal muscle. Acta Physiol. Scand..

[25] Coh M., Peharec S., Bacic P., Kampmiller T. (2009). Dynamic factors and electromyographic activity in a sprint start. Biol. Sport.

[26] Leys C., Leys C., Klein O., Bernard P., Licata L. (2013). Detecting outliers: Do not use standard deviation around the mean, use absolute deviation around the median. J. Exp. Soc. Psychol..

[27] Ratcliff R. (1993). Methods for dealing with reaction time outliers. Psychol. Bull..

[28] Klapp S.T. (1995). Motor response programming during simple and choice reaction time: The role of practice. J. Exp. Psychol. Hum. Percept. Perform..

[29] Klapp S.T., Jagacinsky R.J. (2011). Gestalt principles in the control of motor actions. Psychol. Bull..

[30] Kovacs A.J., Muhlbauer T., Shea C.H. (2009). The coding and effector transfer of movement sequences. J. Exp. Psychol. Hum. Percept. Perform..

[31] Porter J.M., Wu W.F.W., Partridge J.A. (2010). Focus of attention and verbal instructions: Strategies of elite track and field coaches and athletes. Sport Sci. Rev..

